# Distinct Molecular Landscape of Epstein–Barr Virus Associated Pulmonary Lymphoepithelioma-Like Carcinoma Revealed by Genomic Sequencing

**DOI:** 10.3390/cancers12082065

**Published:** 2020-07-27

**Authors:** Shuk-Ling Chau, Joanna Hung-Man Tong, Chit Chow, Johnny Sheung-Him Kwan, Raymond Wai-Ming Lung, Lau-Ying Chung, Edith Ka-Yee Tin, Shela Shu-Yan Wong, Alvin Ho-Kwan Cheung, Rainbow Wing-Hung Lau, Calvin Sze-Hang Ng, Tony Shu-Kam Mok, Kwok-Wai Lo, Ka-Fai To

**Affiliations:** 1 Department of Anatomical and Cellular Pathology, State Key Laboratory of Translational Oncology, The Chinese University of Hong Kong, Hong Kong, China; shukling@cuhk.edu.hk (S.-L.C.); jtong@cuhk.edu.hk (J.H.-M.T.); chit@cuhk.edu.hk (C.C.); shkwan@cuhk.edu.hk (J.S.-H.K.); Raymond_lung@cuhk.edu.hk (R.W.-M.L.); lauying.chung@yahoo.com.hk (L.-Y.C.); Edithtin@yahoo.com.hk (E.K.-Y.T.); shela@cuhk.edu.hk (S.S.-Y.W.); cheung_hokwan@hotmail.com (A.H.-K.C.); kwlo@cuhk.edu.hk (K.-W.L.); 2 Sir Y.K. Pao Cancer Center, The Chinese University of Hong Kong, Hong Kong, China; 3 Division of Cardiothoracic Surgery, Department of Surgery, The Chinese University of Hong Kong, Hong Kong, China; rainbowlau@surgery.cuhk.edu.hk (R.W.-H.L.); calvinng@surgery.cuhk.edu.hk (C.S.-H.N.); 4 Department of Clinical Oncology, The Chinese University of Hong Kong, Hong Kong, China; tony@clo.cuhk.edu.hk

**Keywords:** lymphoepithelioma-like carcinoma, non-small cell lung cancer, genomics, next-generation sequencing

## Abstract

Pulmonary lymphoepithelioma-like carcinoma (LELC) is a subtype of non-small cell lung cancer (NSCLC) characterized by marked lymphocytic infiltration and association with Epstein–Barr virus (EBV). The molecular basis underlying the disease remains unclear. We sought to study the molecular landscape by multiple approaches including whole genomic sequencing, capture-based targeted sequencing, fluorescent in situ hybridization and immunohistochemistry. Tumor cells from 57 EBV-positive pulmonary LELCs were isolated by careful microdissection prior to genomic sequencing. Integrated analysis revealed a distinct genomic landscape of low *TP53* mutation rate (11%), low incidence of known drivers in the RTK/RAS/RAF (11%) and PI3K/AKT/mTOR pathways (7%), but enriched for loss-of-function mutations in multiple negative regulators of the NF-κB pathway. High level programmed cell death ligand-1 (PD-L1) expression was shown with 47% and 79% of the cases showing positive PD-L1 immunoreactivity at ≥50% and ≥1% tumor proportion score, respectively. Subsets of the patients with actionable fibroblast growth factor receptor 3 (*FGFR3)* aberrations (4%) and mismatch repair deficiency (4%) were potentially eligible for precision medicine. Pulmonary LELC showed a distinct genomic landscape, different from major NSCLC subtypes but resembled that of EBV-associated nasopharyngeal carcinoma. Our work facilitated the understanding of molecular basis underlying pulmonary LELC to explore potential therapeutic options.

## 1. Introduction

Lung cancer is the leading cause of cancer-related death worldwide, causing an estimated 1.8 million deaths in 2018 [1]. Pulmonary lymphoepithelioma-like carcinoma (LELC) is a subtype of non-small cell lung cancer (NSCLC), accounting for 0.9% of lung cancer. It is more prevalent in South-East Asia [2]. First described in 1987, LELC has a distinct morphological feature of undifferentiated carcinoma with a typical syncytial growth pattern, large vesicular nuclei with prominent nucleoli, and heavy infiltration of lymphocytes [3]. It is characterized by Epstein–Barr virus (EBV) infection, and morphologically resembles nasopharyngeal carcinoma (NPC), an epithelial malignancy with EBV infection. Pulmonary LELC shows favorable outcomes in comparison with other non-LELC NSCLC subtypes [4].

The primary treatment of pulmonary LELC is radical resection at an early stage and multimodal therapy, including chemotherapy and radiotherapy, at an advanced stage [5]. Previous molecular studies have shown that major driver events, i.e., *EGFR*, *KRAS*, *MET*, *ALK* and *ROS1*, are rare in pulmonary LELC [5,6,7,8,9,10]. Therefore, pulmonary LELC patients may not benefit from personalized targeted therapies commonly used for NSCLC. Comprehensive molecular profiling might reveal novel actionable molecular targets to guide the treatments of pulmonary LELC.

Extensive lymphoplasmacytic infiltrates in LELC challenge the precise molecular profiling. Our study aimed to investigate the molecular background of a large cohort of EBV-associated pulmonary LELC that has undergone meticulous microdissection to enrich tumor content. Integrated analysis of whole genome sequencing and capture-based targeted sequencing could provide comprehensive genomic data with increased depth and breadth. Our work provides new insights into the molecular mechanisms underlying pulmonary LELC and therapeutic strategies for the disease.

## 2. Result

### 2.1. Patient Cohort Characteristics and Survival Analysis

**Figure 1:**
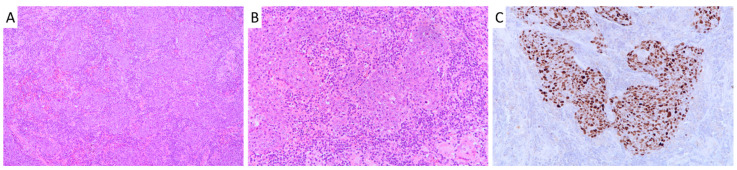
Histology of pulmonary lymphoepithelioma-like carcinoma. (**A**) and (**B**) Hematoxylin and eosin staining of pulmonary LELC showing large tumor cells intermixed with extensive lymphocytes. (Original magnification ×100 in A; ×200 in B). (**C**) Presence of Epstein–Barr virus in pulmonary LELC shown by in situ hybridization of *Epstein–Barr virus-encoded small RNAs* (Original magnification ×200). LELC, lymphoepithelioma-like carcinoma.

The study cohort consisted of 57 pulmonary LELC patients with a median age of 54 years old (range: 37–79) at the time of diagnosis. The female to male ratio was 1.2:1. Seventeen (35%) patients had a history of smoking. Fifteen (26%), 20 (35%), 20 (35%) and 2 (4%) patients were diagnosed at stage I, II, III and IV, respectively (Table 1). The majority of the tumors (56/57, 98%) were positive for squamous cell marker p40 while 93% (53/57) were negative for thyroid transcription factor-1 (TTF-1) (Table S1), suggesting a squamous cell lineage. All cases were positive for EBV infection as demonstrated by *Epstein–Barr virus-encoded small RNAs* (*EBERs*) in situ hybridization (ISH) as shown in Figure 1.

Follow-up information was available in 54 patients with a median follow-up time of 60 months (range 5 to 275 months). By univariate analysis, lymph node metastasis and advanced stage were significantly associated with worse survival with a hazard ratio (HR) of 3.97 (95% confidence interval (CI) 1.59–9.94, *p* = 0.003) and 4.24 (95% CI 1.57–11.41, *p* = 0.004), respectively (Table S2). Other clinicopathological parameters including age, gender, smoking history and tumor size did not associate with patient survival.

To compare the clinicopathological features of pulmonary LELC with other histological subtypes of NSCLC, a consecutive cohort of surgically resected primary NSCLC (*n* = 933) between 1996 and 2016 was retrieved. This cohort consisted of 572 adenocarcinomas, 259 squamous cell carcinomas, 30 large cell carcinomas, 38 adenosquamous carcinomas, and 34 sarcomatoid carcinomas. Compared with NSCLC of other histological subtypes, pulmonary LELC was associated with younger age (LELC vs. non-LELC, 55 vs. 65 years, *p* < 0.001), female (LELC vs. non-LELC, 54% vs. 34%, *p* = 0.002), never-smokers (LELC vs. non-LELC, 65% vs. 36%, *p* < 0.001) and larger tumor size (LELC vs. non-LELC, 4.7cm vs. 4.1cm, *p* = 0.045) (Table S3). Patients with pulmonary LELC showed a significantly better survival (*p* = 0.024) than those with other subtypes of NSCLC. Male gender (*p* = 0.04), larger tumor size (*p* < 0.001), lymph node metastasis (*p* < 0.001), advanced stage (*p* < 0.001), large cell carcinoma (*p* = 0.031) and sarcomatoid (*p* < 0.001) histology were associated with poorer survival in NSCLC patients. Pulmonary LELC, together with gender, stage and sarcomatoid histology subtype remained independent prognostic factors by multivariate analysis (Table S3).

### 2.2. Mutational Signatures, Structural Variations and Copy Number Variations in Pulmonary LELC by Whole Genome Sequencing

**Figure 2:**
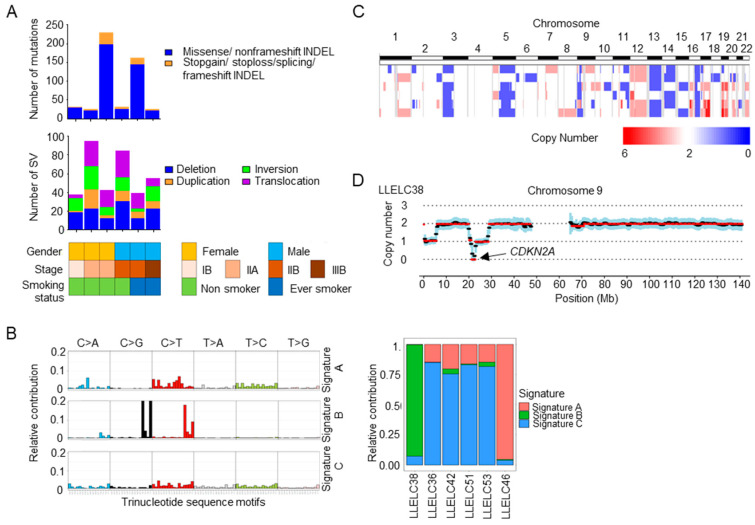
Frequencies of non-silent somatic mutations and structure variations, mutational signatures and copy number alterations of pulmonary lymphoepithelioma-like carcinoma identified by whole genomic sequencing. (**A**) Frequencies of non-silent somatic mutations and structural variations in pulmonary LELC. (**B**) (Left) Three mutational signatures A–C were identified. Mutational signatures were displayed according to 96 substitutions classification defined by the substitution class and sequence context immediately before and after the mutated base. (Right) The relative contribution of signatures A–C in six pulmonary LELC tumors. Each bar represents a single pulmonary LELC case. (**C**) Global chromosomal gains (shown in red) and losses (shown in blue) across six pulmonary LELCs. (**D**) Homozygous deletion of *CDKN2A* was identified in one tumor (LLELC38). LELC, lymphoepithelioma-like carcinoma. SV, structural variation.

Whole genome sequencing was performed in six pairs of pulmonary LELCs and the corresponding normal lung tissues (Figure S1). A total of 505 non-silent somatic mutations were identified in 490 genes (Figure 2A). The average non-silent mutation rate was 2.5 mutations per megabase (Mb) (range: 0.8–6.9 mutations per Mb, median: 0.9 mutations per Mb). All non-silent somatic mutations identified are listed in Table S4. The predominant type of substitution was C>T transition. The non-negative matric factorization approach identified three distinct mutational signature patterns (designated as Signature A, B and C in Figure 2B) composed of different single base substitution (SBS) signature profiles as described previously [11]. Signature C, mainly composed of SBS5 and SBS40 (Mutational Signatures V3, COSMIC), was the predominant pattern that was reported to be correlated with patient age (Figure 2B). Signature A and B were present, each in one case. Signature A consisted of SBS6, SBS15, SBS20 and SBS44, indicative of defective DNA mismatch repair. It was present in a pulmonary LELC patient (LLELC46) with a high mutation rate of 5.4 mutations per Mb. Signature B contained SBS2 and SBS13, both of which were attributed to the apolipoprotein B mRNA-editing catalytic polypeptide-like (APOBEC) family of enzymes.

Whole genome sequencing identified a mean of 59 (range 37–95) structural variations per tumor, including 116 deletions, 81 inversions, 52 duplications and 103 chromosomal translocations (Table S5). A large deletion of approximately 3000 base-pair (bp) was detected on chromosome 14q, spanning from intron 2 to intron 3 of *TRAF3*, a negative regulator of the NF-κB pathway. This deletion was validated by targeted sequencing.

Copy number variation analysis revealed common loss of chromosome 3p, 5q, 13q and 16q, and gain of chromosome 12 in pulmonary LELC (Figure 2C). Homozygous loss of *CDKN2A/CDKN2B* locus at chromosome 9p21.3 was identified in one tumor (Figure 2D).

### 2.3. Targeted Sequencing of Pulmonary LELC

Targeted sequencing was performed on six pairs of pulmonary LELCs in the discovery set and an extended validation set of 51 cases, using a panel of 843 cancer-related genes that targeted approximately 5.25 Mb of the human genome (Table S6). Mean sequencing coverage of tumor and normal samples reached 904× and 170×, respectively. In 57 LELCs, 466 nonsilent somatic mutations were identified in 295 genes. The average non-silent mutation rate was 2.8 mutations per Mb (range: 0.3–19.7 mutations per Mb, median: 1.7 mutations per Mb). Somatic mutations and actionable alterations with the OncoKB level of evidence are listed in Tables S7 and S8, respectively [12].

**Figure 3:**
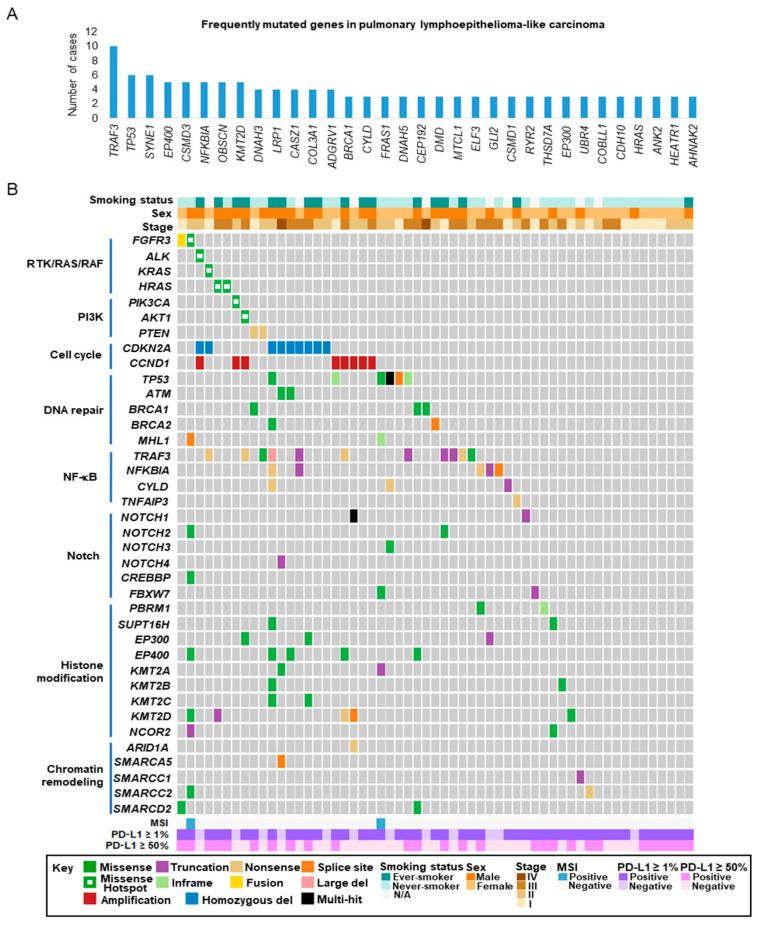
Landscape of molecular alterations in pulmonary lymphoepithelioma-like carcinoma. (**A**) Frequently mutated genes discovered by targeted sequencing. (**B**) Spectrum of key molecular alterations in pulmonary LELC by integrated analysis. LELC, lymphoepithelioma-like carcinoma.

Somatic mutations occurred at a low frequency but were widely distributed in cancer-related genes. Eight top frequently mutated genes were identified in at least five patients: *TRAF3* (18%), *TP53* (11%), *SYNE1* (11%), *EP400* (9%), *CSMD3* (9%), *NFKBIA* (9%), *KMT2D* (9%) and *OBSCN* (9%) (Figure 3A). *TRAF3* and *NFKBIA* are negative regulators of the NF-κB pathway. *TP53* is a tumor suppressor gene for DNA repair and genome integrity. *EP400* and *KMT2D* are involved in histone modifications.

The similarity of mutational landscape between pulmonary LELC and other cancer groups was evaluated by cosine similarity of gene alternation frequency. We employed 93 genes with non-silent single nucleotide variations (SNV) and/or insertion/deletion (indel) identified in at least two pulmonary LELC patients by targeted sequencing. Three published whole exome sequencing datasets with tumor/normal pairs were used for analysis: lung adenocarcinoma (TCGA LUAD), lung squamous cell carcinoma (TCGA LUSQ) and our previous data on nasopharyngeal carcinoma (NPC) [13,14,15]. Using an 80% cutoff for cosine similarity, we found that the mutational landscape of pulmonary LELC was similar to that of NPC, but different from those of lung adenocarcinoma and lung squamous cell carcinoma (cosine similarity, pulmonary LELC vs. NPC 0.83; vs. TCGA LUSQ 0.68; vs. TCGA LUAD 0.68). The finding suggested a higher similarity in mutational profile between pulmonary LELC and NPC.

### 2.4. Actionable Alterations in the RTK/RAS/RAF and PI3K/AKT/mTOR Signaling Pathways

Clinically relevant actionable alterations in the RTK/RAS/RAF and PI3K/AKT/mTOR pathways were detected in a subset of pulmonary LELCs (Figure 3B and Table S8). These included *HRAS* (4%, G13V and Q61R), *KRAS* (2%, G12V), *PIK3CA* (2%, E545K) and *AKT1* (2%, E17K). Notably, we detected an activating mutation of *ALK* (2%, R1275Q) and actionable alterations in *FGFR3* (4%, *FGFR3* R248C and *FGFR3*-*TACC3* fusion). The mutations in *HRAS*, *KRAS, PIK3CA, AKT1*, *ALK* and *FGFR3* were confirmed by Sanger sequencing. *FGFR3* gene rearrangement was validated by RNAseq. The genomic breakpoint of *FGFR3*-*TACC3* and the fusion transcript are shown in Figure S2. Loss-of-function mutations were found in a tumor suppressor gene, *PTEN* (4%). Actionable alterations were commonly found in adenocarcinoma (*EGFR* activating mutation, *ALK* translocation and *ROS1* translocation) and squamous cell carcinoma (*FGFR1* amplification, *PIK3CA* amplification and *SOX2* amplification), but were not detected in pulmonary LELC. The overall mutation rate was 11% in the RTK/RAS/RAF and 7% in the PI3K/AKT/mTOR pathway.

### 2.5. Alterations in Cell Cycle Regulatory Genes

**Figure 4:**
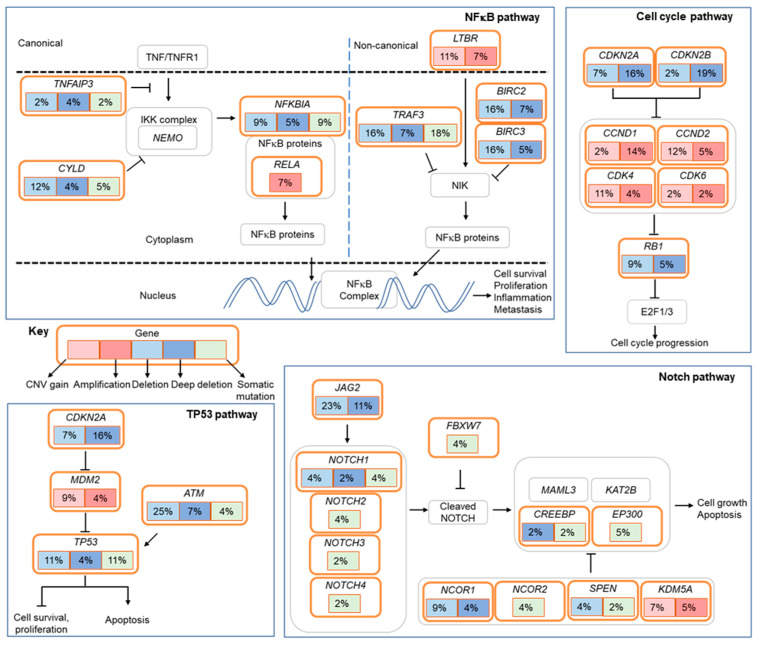
Altered pathways in pulmonary lymphoepithelioma-like carcinoma. Frequencies (%) of genetic alterations for pulmonary LELC tumors were shown. Alterations were defined as somatic mutations, amplifications and deletions affecting the TP53, cell cycle, NF-κB and Notch pathways. Amplification of *CCND1* and homozygous deletion of *CDKN2A* were determined by fluorescence in situ hybridization; amplification and deletion of other genes by targeted sequencing. LELC, lymphoepithelioma-like carcinoma.

Dysregulation of the cell cycle was implicated in pulmonary LELC (Figure 4). Homozygous loss of *CDKN2A* was detected by whole genome sequencing and validated by fluorescence in situ hybridization (FISH) (Figure S3). FISH analysis revealed 16% and 14% of pulmonary LELC harbored homozygous loss of *CDKN2A* and high amplification of *CCND1*, respectively. In addition, deletion of *RB1* and amplification of cyclins, such as *CDK4* and *CDK6*, occurred in pulmonary LELC. These events could lead to cell cycle progression.

### 2.6. Loss-of-Function Mutations in Negative Regulators of The NF-κB Pathway

Another prominently altered pathway in pulmonary LELC was the NF-κB pathway (Figure 4). Multiple loss-of-function mutations were identified in negative regulators of the NF-κB pathway, namely *TRAF3*, *CYLD*, *NFKBIA* and *TNFAIP3*. *TRAF3* was mutated at 18% of pulmonary LELC. The *TRAF3* mutations were predominantly truncating mutations, including nonsense and frameshift mutations (8/10, 80%). The mutation rates of *CYLD*, *NFKBIA* and *TNFAIP3* were 5% (3/57), 9% (5/57) and 2% (1/57), respectively, and all the mutations were truncating mutations (Figure S4). Somatic alterations of negative regulators in the NF-κB pathway were detected in 30% of pulmonary LELC. Additional lesions included *LTBR* amplification and *RELA* amplification.

**Figure 5:**
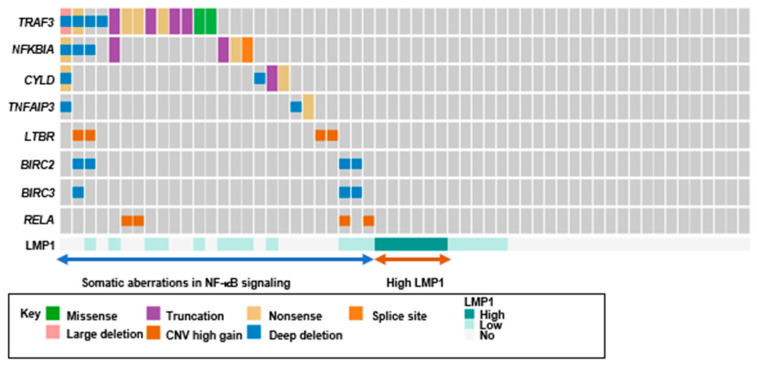
Genetic alterations altered in the NF-κB pathway and expression of viral protein LMP1 in pulmonary lymphoepithelioma-like carcinoma. Genomic alterations in the NF-κB pathway and high expression of LMP1 occurred mutually exclusively in pulmonary LELC. LELC, lymphoepithelioma-like carcinoma; LMP1, latent membrane protein 1. CNV, copy number variation.

Pulmonary LELC is an EBV-associated malignancy. The viral products play a role in tumorigenesis. The EBV oncoprotein latent membrane protein 1 (LMP1) serves as a constitutive activator of the NF-κB pathway. We evaluated the expression of two EBV viral proteins, latent membrane protein 1 (LMP1) and latent membrane protein 2A (LMP2A), by immunohistochemistry. Positive expression of LMP1 and LMP2A were observed in 42% (24/57) and 12% (7/57) of LELC, respectively (Figure S5). High level LMP1 expression, detected in 11% (6/57) of the tumors, were mutually exclusive to NF-κB somatic alterations (*p* = 0.02, 1-sided Fisher’s exact test) (Figure 5).

### 2.7. Alterations in Epigenetic Regulators

Pulmonary LELC showed widespread alterations in epigenetic regulators. We identified a total of 34 altered genes affecting histone modification in 26 cases, and 11 genes involving chromatin remodeling in 9 cases (Figure 3 and Table S7). Mutations of *KMT2D* and *EP400* were frequently detected and each was found to be mutated in 9% of pulmonary LELC. Multiple cases harbored mutations in a group of histone methyltransferases, including *KMT2A*, *KMT2B* and *KMT2C*.

### 2.8. Alterations in the Notch and TP53 Pathways

Somatic alterations were observed in the Notch signaling pathway (Figure 4). Loss-of-function mutations were present in key players such as *NOTCH1*, *NOTCH4* and *FBXW7* in 7% of the cases. Deletion of *JAG2*, one of five Notch ligands, was detected in 11% of the patients.

Tumor suppressor protein p53 is essential for the maintenance of genome integrity. *TP53* mutations occurred in 11% of pulmonary LELC. Missense mutations and non-frameshift substitutions mainly occurred at the DNA binding domain of *TP53* (Figure 3 and Table S7). Amplification of *MDM2*, a negative regulator of p53, and deletion of *ATM*, one of the master regulators of DNA damage response, occurred in 11% of pulmonary LELC patients (Figure 4).

### 2.9. Microsatellite Instability (MSI) in Pulmonary LELC

The whole genome mutational signature of a pulmonary LELC (LLELC46) suggested a defective DNA mismatch repair pathway. By using MSIsensor, a program that reports the percentage of instable microsatellites as a score, we determined the MSI status of 57 LELCs from targeted sequencing data. Two samples were classified as microsatellite instable (LLELC18 and LLELC46) (Figure S6). Both tumors harbored somatic mutations at the *MLH1* gene (Table S7). We examined the expression of four mismatch repair proteins (MLH1, PMS2, MSH2 and MSH6) by immunohistochemistry (IHC) in LELCs. Both cases showed concurrent loss of MLH1 and PMS2 expression (Figure S7), while the remaining LELCs retained the protein expression of all four mismatch repair proteins. Consistent with the MSIsensor report based on targeted sequencing data, two out of 57 (4%) LELCs showed mismatch repair deficiency demonstrated by loss of mismatch repair protein expression.

### 2.10. Programmed Cell Death Ligand-1 (PD-L1) Expression in Pulmonary LELC

Since immunotherapy becomes a promising therapeutic approach in NSCLC, we evaluated PD-L1 expression in pulmonary LELCs (*n* = 57) using PD-L1 IHC 22C3 pharmDx. The positive rates were 79% and 47% at tumor proportion score (TPS) cut-offs of ≥1% and ≥50%, respectively. PD-L1 expression was not significantly associated with any clinicopathological parameters, including age, sex, smoking history, and stage (*p* > 0.05), and overall survival (TPS ≥ 1%: log rank = 0.70; TPS ≥ 50%: log rank = 0.36).

## 3. Discussion

Pulmonary LELC is a subtype of NSCLC that is characterized by EBV infection and heavy lymphocytic infiltrate. In the present study, we reported the molecular profiling of the first and largest cohort of microdissected pulmonary LELC using whole genome sequencing and targeted sequencing, and the assessment of PD-L1 expression. A subset of pulmonary LELC harbored clinically relevant actionable alterations in the RTK/RAS/RAF and PI3K/AKT/mTOR pathways. Enrichment of somatic alterations implicated aberrant signaling in the cell cycle, NF-κB, Notch and TP53 pathways. In addition, PD-L1 expression was prevalent in pulmonary LELC.

The findings of a distinct genomic background provided insight into how the genetics of pulmonary LELC varied from other NSCLC subtypes. In concordance with previous studies, pulmonary LELC showed a low degree of somatic mutation rate [9,10]. Its median mutation rate was 0.9 and 1.7 mutations per Mb by whole genome sequencing (78×) and targeted sequencing (907×), respectively. Whereas adenocarcinoma and squamous cell carcinoma of lung exhibited higher somatic mutation rates of 6.9 and 8.4 mutations per Mb, respectively [13,14]. Genetic alterations and altered pathways in pulmonary LELC were different from those of other major NSCLC subtypes. Pulmonary LELC tumors expressed p40, a marker that is often positive in tumors of squamous cell origin. However, their genomic profiling lacked diverse genetic alterations characterized in squamous cell carcinoma, such as *SOX2* amplification in the squamous differentiation pathway; *PIK3CA* amplification in the PI3K signaling; and *FGFR1* amplification in the RTK signaling pathway. Concordant with previous molecular studies, major driver events in adenocarcinoma, namely *EGFR* activating mutations, *ALK* gene rearrangement and *ROS1* gene rearrangement, were rarely present in pulmonary LELC, indicating that they were less important events in the pathogenesis of pulmonary LELC [6,16,17]. Our findings revealed pulmonary LELC as a distinct subtype of NSCLC with different genomic features, in addition to its unique histological appearance and persistent EBV infection (Figure 1).

It was important to examine molecular features of pulmonary LELC, as this might unleash the potential of precision medicine for advanced pulmonary LELC. This study identified a subset of pulmonary LELC harboring clinically actionable alterations in the RTK/RAS/RAF and PI3K/AKT/mTOR signaling pathways. Interestingly, it was the first study to identify actionable alterations of *FGFR3* in 4% of pulmonary LELC. Actionable *FGFR3* gene fusions were found to be relatively common in glioblastoma and bladder cancer [18]. Recurrent *FGFR3*-*TACC3* fusions were reported in 2.5% of NPC [19]. However, *FGFR3* alterations were rarely observed in NSCLC. *FGFR3* hotspot mutations, R248C and S249C, and *FGFR3* fusions were found in 0.1% and 0.14% of NSCLC, respectively [20]. *FGFR* fusions were detected in 0.1% and 0.6% of adenocarcinoma and squamous cell carcinoma, respectively [21]. Tumors with *FGFR3* fusions were sensitive to FGFR inhibition [22]. With the prevalence of *FGFR3* at 4%, *FGFR3* aberrations might represent an opportunity for targeted therapy in pulmonary LELC. Clinical trials using FGFR inhibitors may be warranted for LELC patients harboring the *FGFR3* aberrations.

NF-κB regulates diverse biological processes, including immunological functions. It is recognized as a crucial player in cancer initiation and progression [23]. In line with our findings, Hong et al. recently reported that somatic aberrations of *TP53* and multiple negative regulators including *TRAF3*, *CYLD* and *NFKBIA* were commonly detected in pulmonary LELC, suggesting their important roles in this unique cancer type [10]. A similar percentage of LMP1 expression and its mutually exclusive relationship with somatic alterations of the components of the NF-κB pathway were also shown in both studies. Notably, both the present study and the previous report observed the enrichment of genetic aberrations in the multiple negative regulators of NF-κB, which have been reported as a genetic feature of the dysregulated NF-κB pathway in a recent genomic study of NPC [15]. In fact, Hong et al. identified *TRAF3* as one of the frequently mutated genes and Xie et al. reported that *NFKBIA* mutations were frequently detected in pulmonary LELC [9,10]. Intriguing, we revealed multiple loss-of-function mutations in *TRAF3*, *CYLD*, *NFKBIA* and *TNFAIP3.* Such a feature of multiple loss-of-function mutations of NF-κB pathway regulators, leading to activation of NF-κB pathway, has been recently reported in NPC [15,24]. The dysregulation of NF-κB signaling, relating to viral oncoprotein LMP1 and genetic alterations of NF-κB regulators, was a crucial event in NPC tumorigenesis [25,26]. Our findings unveiled the genetic lesions regarding NF-κB and implicated the involvement of the NF-κB pathway underlying the pathogenesis of the disease. The uniqueness of loss-of-function mutations of NF-κB negative regulators and an association of EBV infection suggested a resemblance between pulmonary LELC and NPC in pathogenesis of the disease.

Our study showed an obviously higher mutation rate (17/57, 30%) of these NF-κB negative regulators, especially *TRAF3* (10/57; 18%). In the report of Hong et al., somatic mutations of *TRAF3* were detected in 5% of their cases [10]. They employed different methodologies, including whole exome sequencing (100× coverage), targeted sequence (170× or 300× coverage) and single-nucleotide polymorphism (SNP) arrays to examine the mutational landscape of pulmonary LELC. In general, they found a low somatic mutation rate and revealed the genetic lesions of NF-κB largely owing to copy number variations. Instead of mutations, the group focused on the copy number variations detected by SNP arrays in their report. The low mutation rate of the critical genes may be due to the extensive infiltrating stromal cells/lymphocytes in these tumors (as shown in Figure 1) that might hinder the detection of mutations. In our study, we performed meticulous microdissection to enrich the tumor cell content in each tumor. Furthermore, “deep” targeted sequencing of approximately 900× was performed on the microdissected tumors in order to evaluate somatic alterations of cancer-related genes to minimize the effect of abundant lymphocytic infiltrates in the tumor. These approaches allowed us to detect higher frequencies of somatic mutations in key cancer genes, including *TP53* (11%), *SYNE1* (11%), *EP400* (9%), *CSMD3* (9%), *NFKBIA* (9%), and *KMT2D* (9%).

Furthermore, several important somatic alterations found in the current study have not been reported in LELC before. The actionable *FGFR3* alterations including a hotspot mutation and *FGFR3-TACC3* gene fusion were detected by our “deep” targeted capture sequencing. In addition to the high coverage, our custom-designed 843-gene panel was constructed based on the findings from a previous NPC genomic study and the discovery cohort of six pulmonary LELC cases in this study [15]. The capture probes were empirically designed to include both exon and intron regions of genes with recurrent structural variations, such as *TRAF3, CYLD*, *ALK*, *FGFR2*, *FGFR3*, *ROS1* and *RET*. The unique gene panel design and high coverage deep targeted sequencing allows us to detect the important druggable targets, such as *FGFR3-TACC3* and other rare mutations in pulmonary LELC.

Personalized medicine is revolutionizing the treatment of NSCLC. The use of specific targeted therapy includes the selective tyrosine kinase inhibitors for the treatment of NSCLC with *EGFR* mutation, *ALK* rearrangement or *ROS1* rearrangement. Pulmonary LELC showed genetic features largely distinct from major NSCLCs. Low incidence of clinical actionable alterations in the RTK/RAS/RAF and PI3K/AKT/mTOR pathways indicated that the subtype might not be beneficial to the conventional targeted therapy of NSCLC. As shown in our study, the detection of somatic alterations of various double strand DNA repair genes including *ATM*, *BRCA1* and *BRCA2* (7/57, 12.3%, Figure 3B) suggested a subgroup of patients might be sensitive to PARP1 inhibitor treatment. In addition, the genetic background and histopathological features of pulmonary LELC suggested that it is a disease entity with resemblance to nasopharyngeal carcinoma, an EBV-associated malignancy occurring in the nasopharynx. The treatment advanced in NPC might provide a therapeutic opportunity for pulmonary LELC. Various ongoing preclinical studies on in vitro and in vivo EBV-positive NPC models may provide important information for planning clinical trials of NPC and pulmonary LELC. Therapies for targeting EBV have been explored for potential treatment of NPC [27]. Inhibition of DNA binding capacity of EBV viral protein Epstein–Barr nuclear antigen 1 (EBNA1) has been shown to suppress the growth of EBV-positive xenograft models [28]. VK-2019, an oral EBNA-1 targeting agent, has entered a Phase I/IIa clinical trial (NCT03682055) in patients with EBV-positive NPC. In addition, EBV-specific cytotoxic T-lymphocytes (EBV-CTLs) is an alternative approach to target EBV antigen expressed in NPC [29]. A combination of gemcitabine and carboplatin with EBV-CTLs has entered Phase III clinical trial (NCT02578641) for advanced NPC patients. EBV-targeting therapies might be potentially further developed for the treatment of EBV-associated malignances, including EBV-associated pulmonary LELC. The recruitment of pulmonary LELC patients in these ongoing clinical trials will provide new opportunities to cure this rare cancer. Advances in NPC treatments could provide a future therapeutic strategy direction of pulmonary LELC.

Immunotherapy is emerging as a new kind of personalized targeted therapy in NSCLC. The predictive biomarkers are MSI-high/mismatch repair deficiency and programmed death-1 (PD-1)/PDL-1 protein expression [30,31]. Although a previous study showed that none of seven pulmonary LELC patients was MSI-high, we discovered that mismatch repair deficiency was prevalent at 4% (2/57) of pulmonary LELC [32]. A recent KEYNOTE-042 study showed that pembrolizumab was superior to chemotherapy in locally advanced or metastatic NSCLC patients with PD-L1 TPS ≥ 1% [33]. In this study, 79% of pulmonary LELC were positive with PD-L1 at TPS ≥ 1% by the assessment of PD-L1 IHC 22C3. The result was consistent with previous findings of 66–76% of pulmonary LELC patients positive for PD-L1, with the threshold of 5% positively stained tumor cells [6,9,34]. Among case reports of primary pulmonary LELC patients treated with PD-1 inhibitors, two partial responses were noted, and five patients had stable disease [9,35,36,37]. The presence of the mismatch repair deficiency phenotype and high prevalence of PD-L1 in pulmonary LELC might provide a rationale for immunotherapy. Clinical trials would be warranted to evaluate immunotherapy in treating pulmonary LELC patients with positive PD-L1 expression.

We were aware of the limitation of our study due to its retrospective nature. We were unable to provide treatment response data regarding molecular profiling, which hindered us from further evaluation on therapeutic options. Due to the rarity of pulmonary LELC, the genomic study by whole genome sequencing was limited by scarce availability of frozen tumor tissues. However, our study did provide important insight for understanding the molecular basis underlying pulmonary LELC and guiding potential personalized targeting therapies.

## 4. Materials and Methods

### 4.1. Patients

A cohort of 57 patients with primary pulmonary LELC who underwent surgical excision in Prince of Wales Hospital, Hong Kong, during the period of 1995–2019 was recruited. Nasopharyngeal examination had been performed in all patients to exclude metastatic nasopharyngeal carcinoma. Formalin-fixed paraffin-embedded (FFPE) tissue specimens were retrieved from the pathologic archive. Routine hematoxylin and eosin-stained slides for tumor blocks were reviewed by an expert pulmonary pathologist (K.F.T.). Pathological stages were determined according to the 8th edition of American Joint Committee on Cancer tumor-node-metastasis classification system. Patients were categorized into either never-smoker (smoke less than 100 cigarettes in their lifetime) or ever-smoker (smoke more than 100 cigarettes in their lifetime). The study protocol was approved by the Joint CUHK-NTE Clinical Research Ethics Committee, Hong Kong (reference number: 2014.070).

### 4.2. Whole Genome Sequencing (WGS)

Whole Genome Sequencing was performed on six pairs of fresh frozen pulmonary LELCs and corresponding normal lung tissues. Tumor tissues were subjected to laser captured microdissection. Sections of tissues were mounted on Leica PEN-membrane slides and stained with hematoxylin. Laser captured microdissection was performed on a Leica LMD7000 system (Leica Microsystems, Wetzlar, Germary). Regions of tumor cells were selected via Leica LMD software and were isolated by laser cutting. Genomic DNA of isolated tumor cells were purified with QIAamp DNA Micro Kit (QIAGEN, Venlo, The Netherlands).

Illumina TruSeq DNA Sample preparation Kit (Illumina, San Diego, CA, USA) was applied to prepare sequencing libraries of 300–400 bp average insert size. WGS was performed on Illumina HiSeq X platform with a standard 150 bp paired-end read previously described [38]. Normal and tumor samples reached mean target coverage of 55× and 78×, respectively. The raw sequence reads were processed and aligned to the hg19 human reference genome with Issac aligner [39]. Identification of somatic SNV and indel was conducted by Strelka [40] and MuTect [41]. Analysis of structural variation (SV) was performed with Manta [42]. Somatic copy number variation (CNV) was predicted with Sequenza [43]. Somatic CNV, SV and nonsynonymous somatic mutations of each pulmonary LELC were visualized by CIRCOS (Figure S1) [44]. Somatic mutations detected by WGS were verified by targeted sequencing for a total of 81 positions, achieving a verification rate of 94%.

### 4.3. Capture-Based Targeted Sequencing

Capture-based targeted sequencing with a custom-designed 843-gene panel was performed on the DNA from six pairs of pulmonary LELCs in the discovery set and an extended validation set of 51 cases (four fresh frozen and 47 FFPE tissues). All tumors underwent meticulous microdissection either manually under light microscope or by laser captured microdissection. Frozen sample DNA and FFPE DNA were purified with QIAamp DNA Micro Kit and truXTRAC FFPE Kits (Covaris, Woburn, MA, USA), respectively, according to the manufacturer’s protocol. Genomic DNA was sonicated to 200 bp. Libraries were prepared using KAPA HyperPrep kit (KAPA Biosystems, Wilmington, Massachusetts) and enriched with a custom-designed 843-gene solution-based hybrid capture panel (Roche, Basel, Switzerland). This cancer-related gene panel targeted approximately 5.25 Mb of the human genome (Table S6). The libraries were sequenced on Illumina HiSeq platform (Illumina, San Diego, CA, USA) at 150 bp paired-end to achieve a goal coverage of 50× and 500× for normal and tumor samples, respectively. Alignment and variant calling were performed as previously described [45] with slight modifications. In brief, quality-filtered sequencing reads were aligned to the hg 19 human reference genome using BWA [38]. Duplicated reads removal and indel realignment were done with Picard v1.9.7 (http://broadinstitute. github.io/picard/) and GATK v3.6 (Broad Institute, Cambridge, MA, USA) following GATK Best Practice recommendations [46]. Somatic SNV and indels were detected by comparing tumor and matched normal using MuTect2 [47], Vardict [48], VarScan2 [49], FreeBayes [50], SAMtools [51] and Strelka [40]. For the tumor sample without matched normal, potential germline mutations were removed if the estimated minor allele frequency is greater than 1% in public databases. CNV and gene fusions were called by CNVkit [52] and Manta [42], respectively. MSI status was estimated using MSIsensor [53] which computes the percentage of microsatellite loci that are unstable in the targeted regions. MSI-positive tumors were defined as those with the proportion of microsatellite loci showing instability greater than one standard deviation above the mean (Figure S6).

### 4.4. Immunohistochemistry (IHC), Fluorescence in Situ Hybridization (FISH) and in Situ Hybridization (ISH)

IHC and FISH were performed on FFPE tissue as described previously [45]. Antibodies and dilutions, antigen retrieval methods and staining condition of IHC analysis are summarized in Table S9. PD-L1 expression in tumor cells was evaluated by PD-L1 22C3 PharmDx assay (Dako, Agilent Technologies, Santa Clara, CA, USA) using TPS as described previously [54]. The tumor was considered to have PD-L1 expression if TPS ≥ 1% and high PD-L1 expression if TPS ≥ 50%. LMP1 expression was assessed with the HistoScore system that combined the staining intensity (0,1,2,3) and percentage (0–100) of positive cells to assign a score ranging from 0 to 300. High LMP1 expression was defined as HistoScore >100 as previously described [15]. Amplification of *CCND1*, *FGFR1*, *PIK3CA* and *SOX2*, and deletion of *CDKN2A* were assessed by dual-color FISH probes listed in Table S10. *EBERs* ISH was performed with an EBER probe ISH kit (Leica, Newcastle, U.K.) to confirm the presence of EBV in the paraffin sections.

### 4.5. Statistics

Pairwise comparisons among groups were analyzed by chi-square test or Fisher’s exact test for categorical variables, and paired t test for continuous variables. Kaplan–Meier analysis using the log-rank test was employed for survival analysis with comparison among different groups of patients. Cox proportional hazards regression was used for univariate and multivariate survival analyses. Cosine similarity was used to evaluate the similarity of mutational profile between pulmonary LELC and other cancers. All statistical analyses were performed in IBM SPSS Statistics (Version 19.0, Armonk, NY, USA). A 2-tailed *p* value < 0.05 was considered as statistically significant in all tests except for the test of mutually exclusive, in which a 1-tailed *p* value cutoff of 0.05 was used to declare statistical significance.

## 5. Conclusions

In summary, this molecular study of EBV-associated pulmonary LELC revealed a subset of clinically relevant alterations in the RTK/RAS/RAF and PI3K/AKT/mTOR pathways, enrichment of somatic alterations in the NF-κB pathway and cell cycle regulators, and high prevalence of PD-L1 expression. Pulmonary LELC harbored genetic alterations largely distinct from those of major subtypes of non-small cell lung cancer but resembling those of nasopharyngeal carcinoma. Our work provides insights to personalized treatments in pulmonary LELC.

## Figures and Tables

**Table 1:** Clinicopathologic Features of Pulmonary Lymphoepithelioma-Like Carcinoma Cohort.

Clinical Features	Total (*n* = 57)
Age	
Mean	55 years
Median	54 years
Range	37–79 years
Tumor size	
Mean	4.7 cm
Median	4.5 cm
Range	1–12 cm
Sex	
Female	31(54)
Male	26(46)
Smoking status	
Never-smoker	31(65)
Ever-smoker	17(35)
Stage	
I	15(26)
II	20(35)
III	20(35)
IV	2(4)
N status	
N0	33(60)
N1-3	22(40)

## Supplementary Materials

The following are available online at https://www.mdpi.com/2072-6694/12/8/2065/s1, Figure S1: Schematic diagram showing somatic mutations, structural arrangements and copy number variations revealed by whole genome sequencing in six pulmonary lymphoepithelioma-like carcinoma cases. SNV, single nucleotide variation; Mb, megabase; Figure S2: FGFR3-TACC3 fusion identified in pulmonary lymphoepithelioma-like carcinoma. (A) Schematic representation of fusion between exon 17 of FGFR3 and exon 8 of TACC3. (B) Detection of genomic breakdown of FGFR3-TACC3 fusion. (C) FGFR3-TACC3 fusion transcript detected by RNA-seq.; Figure S3: Homozygous deletion of CDKN2A in LLELC38 validated by dual color fluorescence in situ hybridization assay of chromosome 9 probe, centromere 9 (red)/ 9p21.3-CDKN2A (green). No green signal was detected in tumor cells, indicating complete loss of CDKN2A locus in tumor cells.; Figure S4: Schematic representation of negative regulators of the NF-kB pathway with somatic mutation sites mapped to the respective protein domains.; Figure S5: Representative images for immunostaining of latent membrane protein 1 and latent membrane protein 2A in pulmonary LELC cases (Original magnification ×100). LMP1, latent membrane protein 1; LMP2A, latent membrane protein 2A; Figure S6: Microsatellite instability (MSI) status estimated by MSIsensor. Red bars indicated MSI-positive cases; Figure S7: Loss of DNA mismatch repair proteins in LLELC46. Representative images for hematoxylin and eosin, in situ hybridization for Epstein–Barr virus-encoded small RNAs, and immunohistochemistry of MLH1, PMS2, MSH2 and MSH6 (Original magnification ×100). HE, hematoxylin and eosin; EBERs, Epstein–Barr virus-encoded small RNAs., Table S1: Immuno-profiles of 57 Epstein–Barr virus-associated pulmonary lymphoepithelioma-like carcinoma; Table S2: Univariate analysis of overall survival in pulmonary lymphoepithelioma-like carcinoma; Table S3: Clinicopathologic features and overall survival analysis of non-small cell lung cancer; Table S4: Somatic mutations of six paired pulmonary lymphoepithelioma-like carcinoma by whole genome sequencing; Table S5: Structural variations of six paired pulmonary lymphoepithelioma-like carcinoma by whole genome sequencing; Table S6: List of genes in targeted capture panel; Table S7: Somatic mutations of 57 pulmonary lymphoepithelioma-like carcinoma by targeted sequencing; Table S8: Actionable alterations with OncoKB level of evidence in pulmonary lymphoepithelioma-like carcinoma; Table S9: Antibodies and staining conditions for immunohistochemistry; Table S10 List of fluorescence in situ hybridization probes in the study.
