# Supplementary figures and images for: Distinct Molecular Landscape of Epstein–Barr Virus Associated Pulmonary Lymphoepithelioma-Like Carcinoma Revealed by Genomic Sequencing
Source: Cancers (Basel). 2020 Jul 27;12(8):2065. doi: 10.3390/cancers12082065 (PMC7463519; doi:10.3390/cancers12082065)

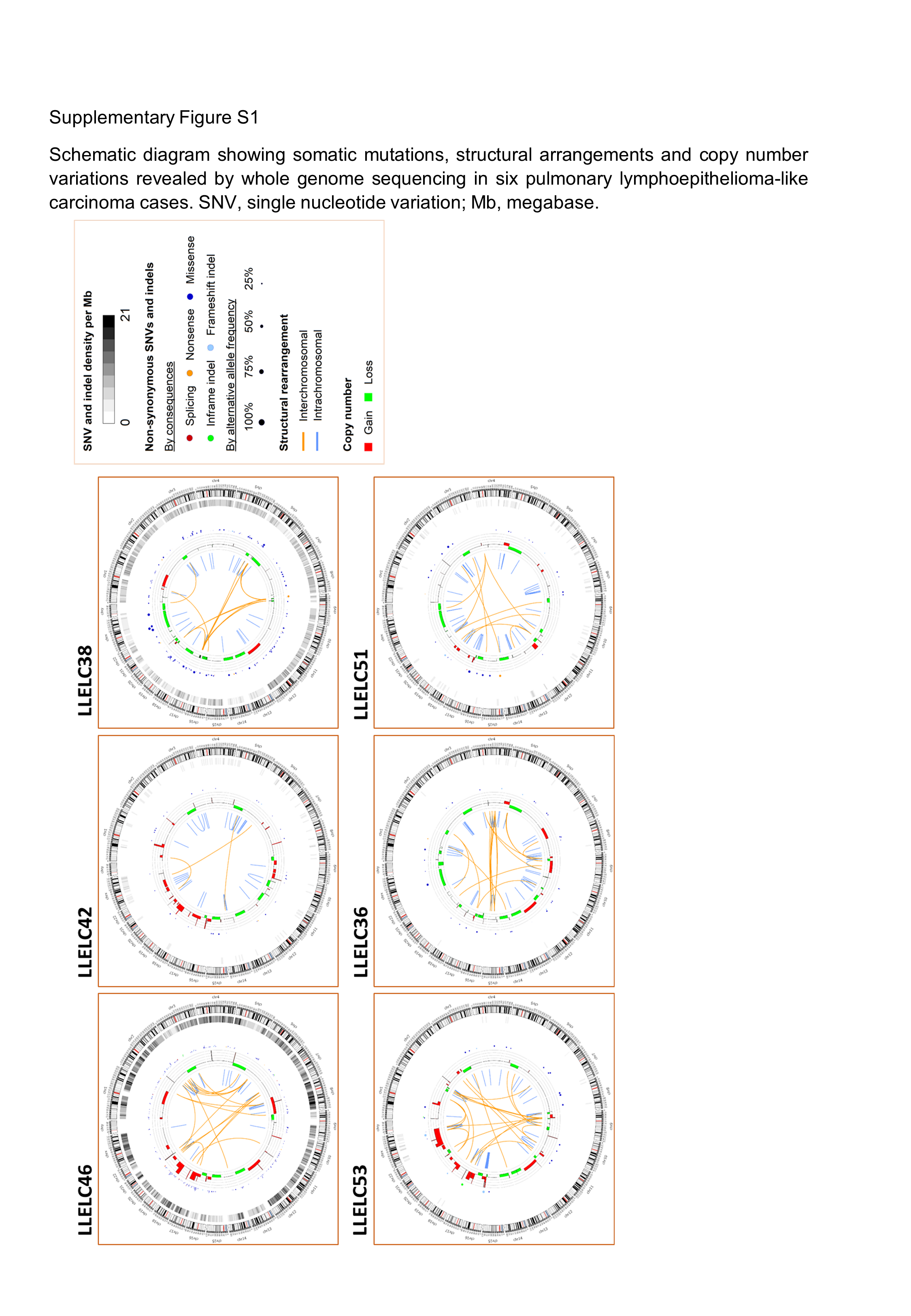

Supplement: Supplementary file 1 [file cancers-12-02065-s001.zip › cancers-870865-supplementary V2/Figure S1.TIF]

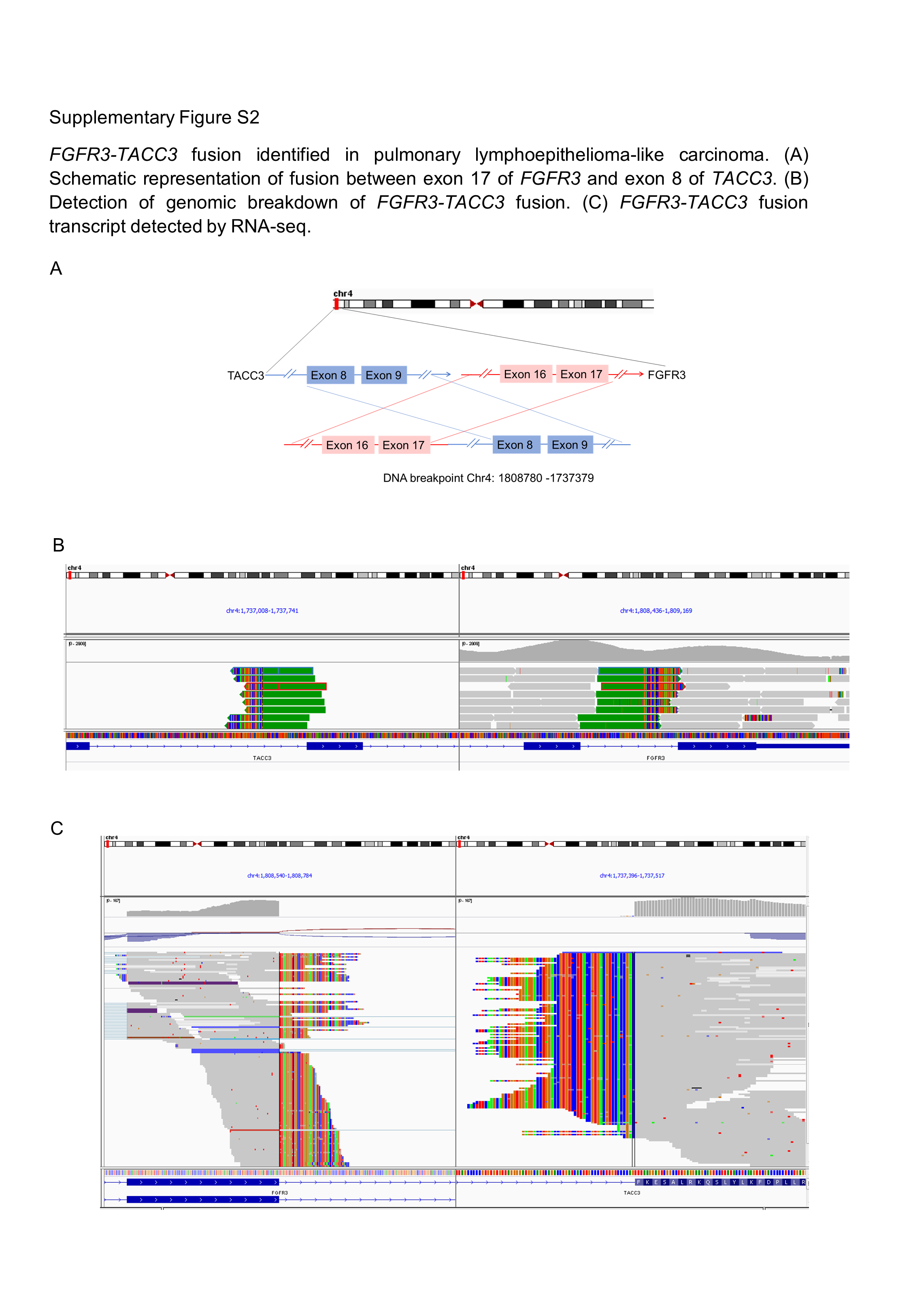

Supplement: Supplementary file 1 [file cancers-12-02065-s001.zip › cancers-870865-supplementary V2/Figure S2.TIF]

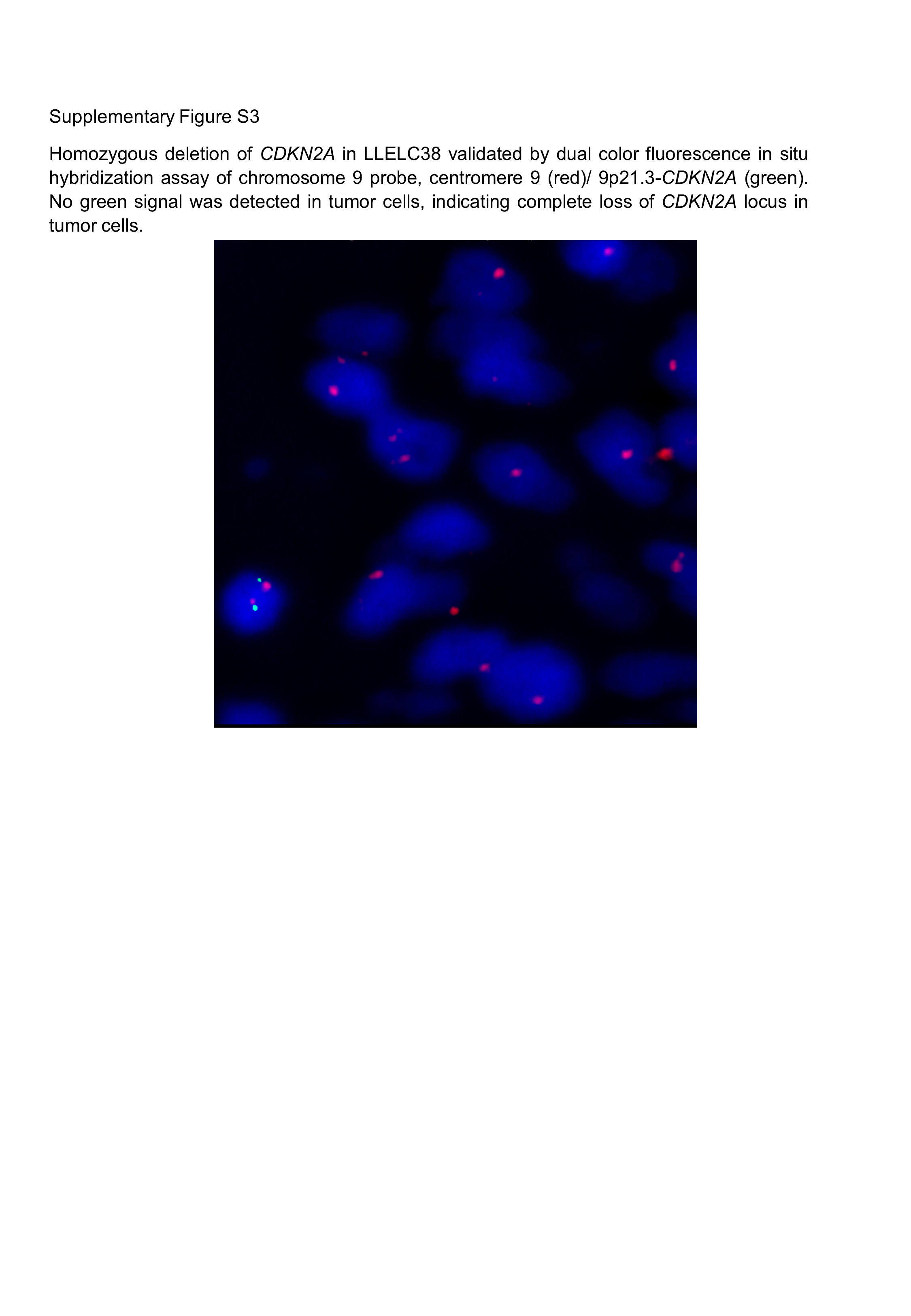

Supplement: Supplementary file 1 [file cancers-12-02065-s001.zip › cancers-870865-supplementary V2/Figure S3.TIF]

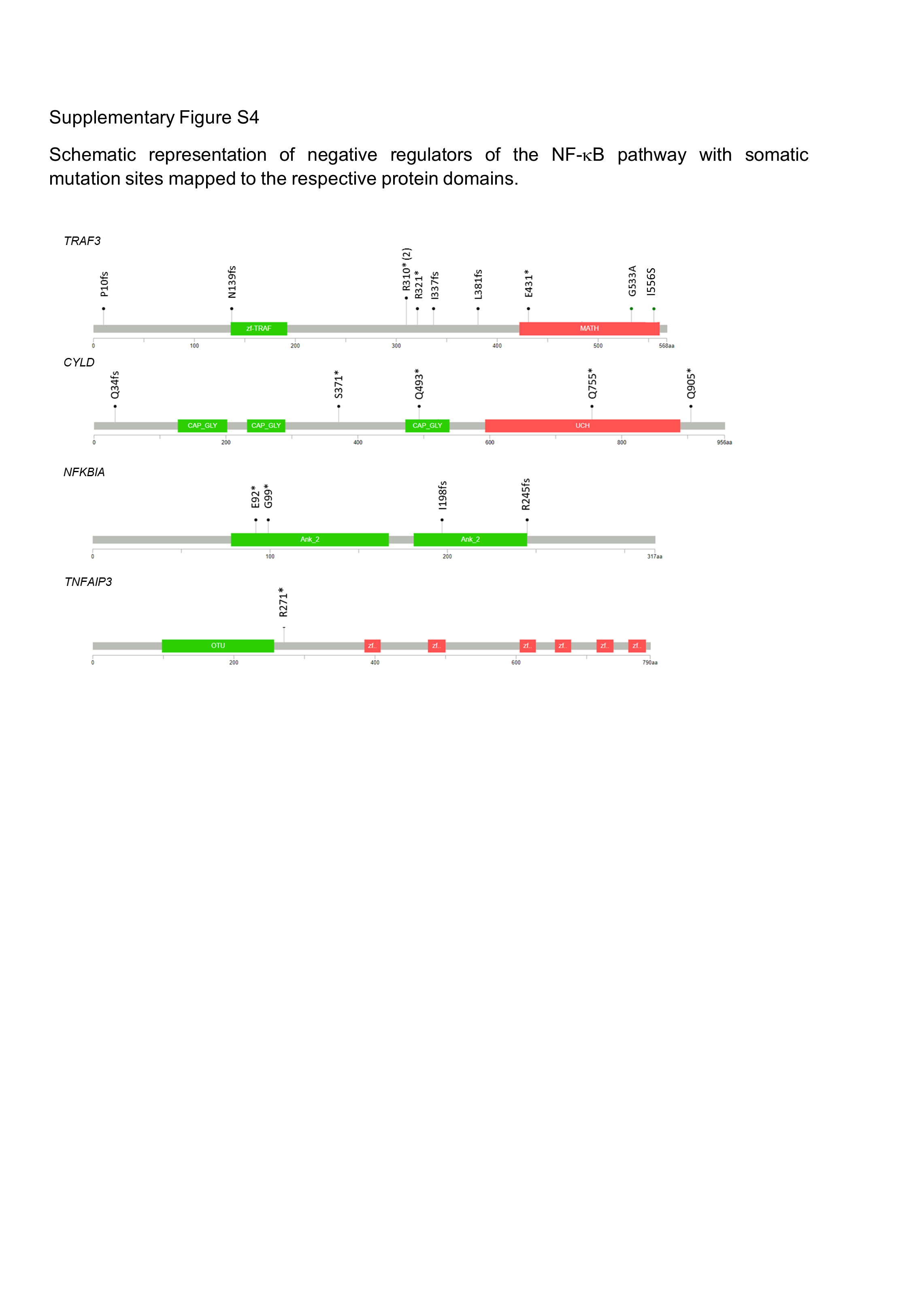

Supplement: Supplementary file 1 [file cancers-12-02065-s001.zip › cancers-870865-supplementary V2/Figure S4.TIF]

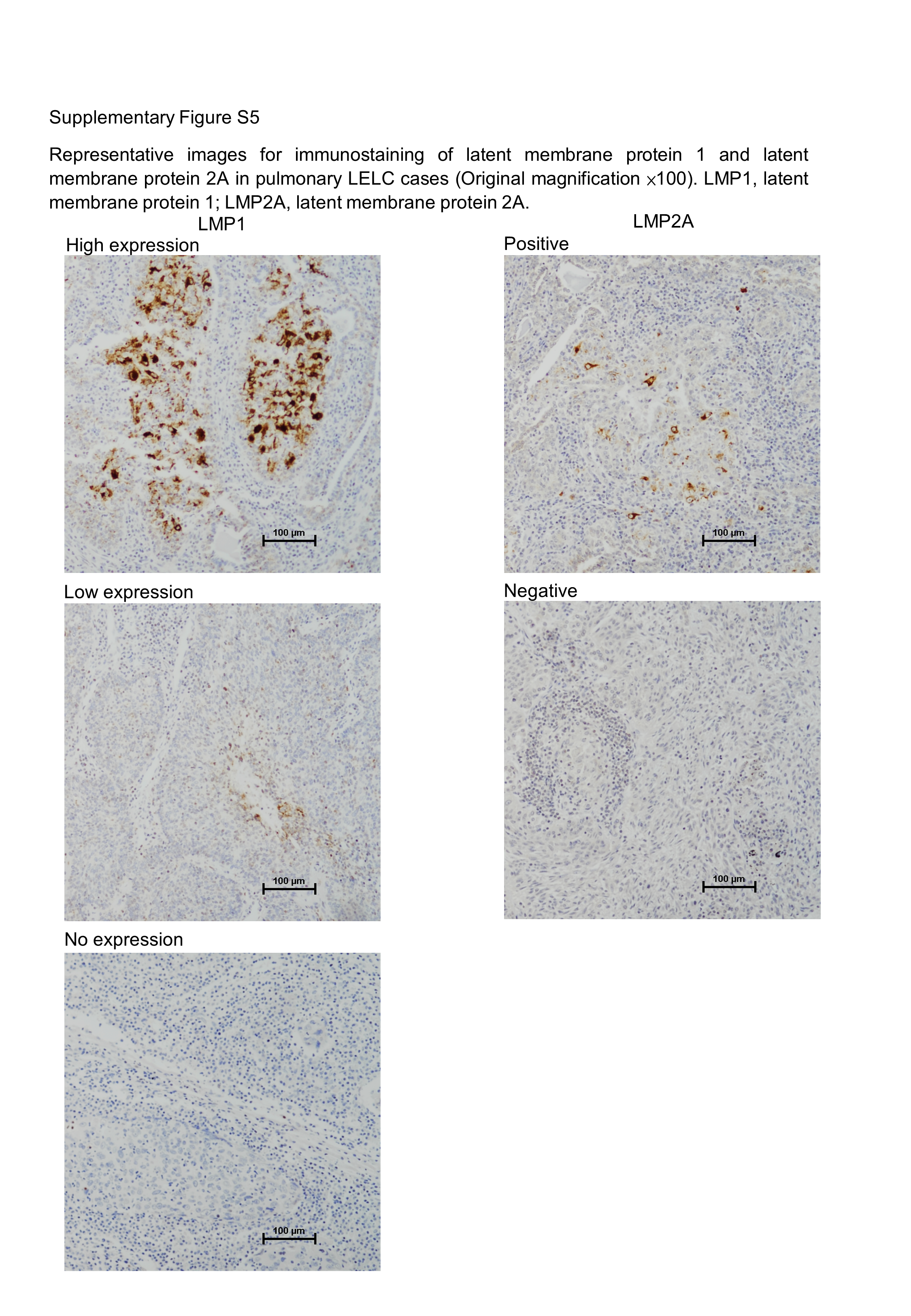

Supplement: Supplementary file 1 [file cancers-12-02065-s001.zip › cancers-870865-supplementary V2/Figure S5.TIF]

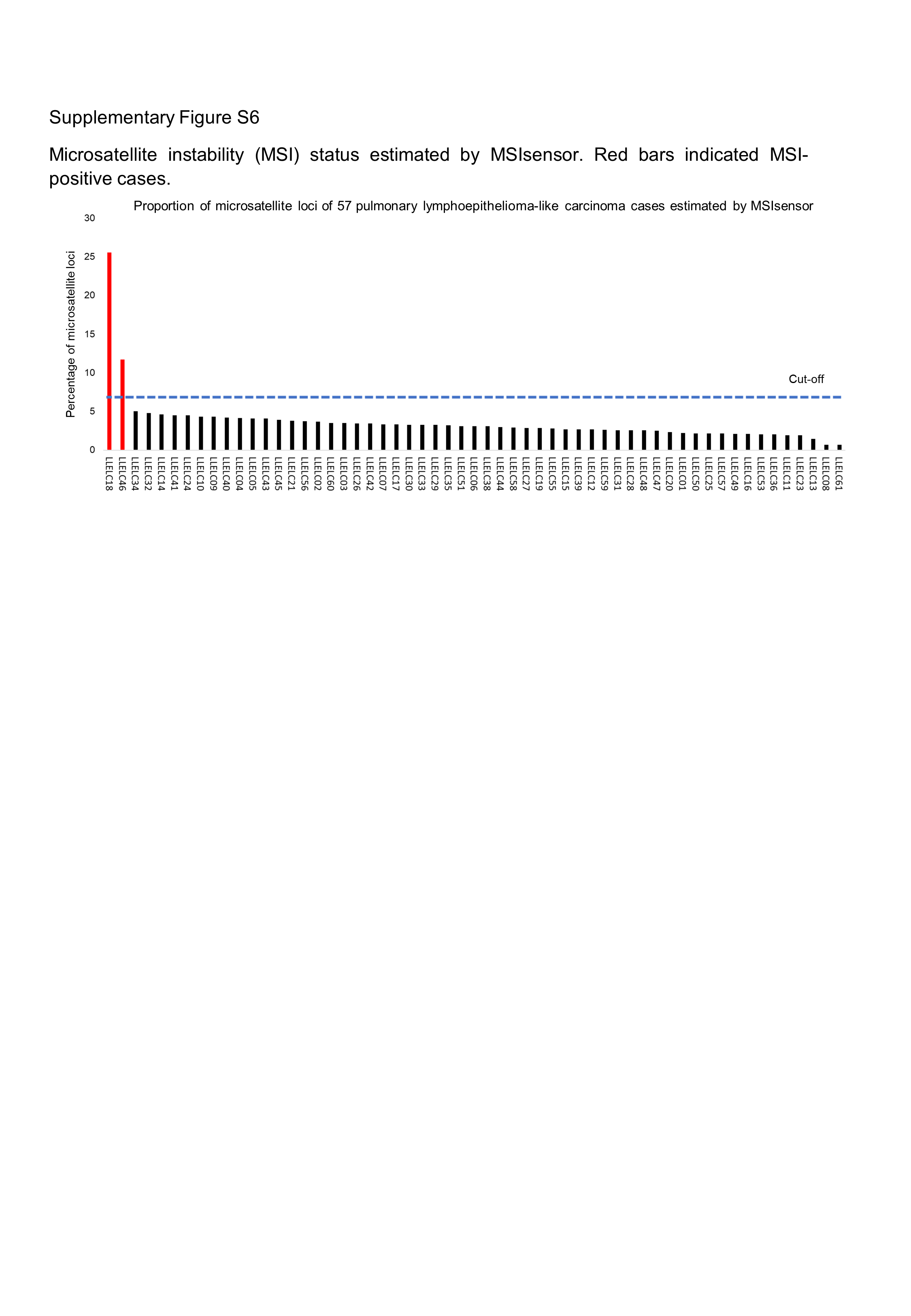

Supplement: Supplementary file 1 [file cancers-12-02065-s001.zip › cancers-870865-supplementary V2/Figure S6.TIF]

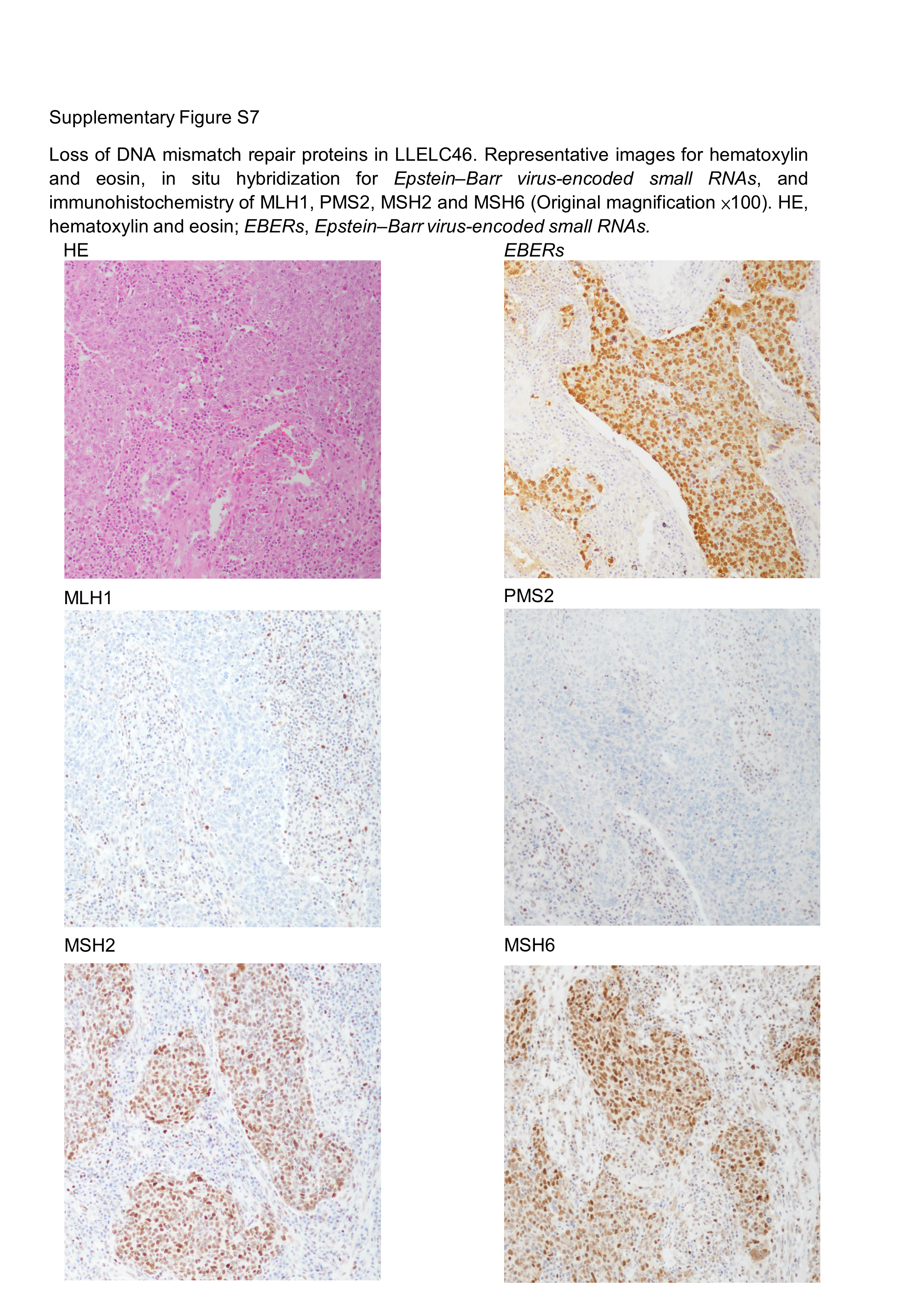

Supplement: Supplementary file 1 [file cancers-12-02065-s001.zip › cancers-870865-supplementary V2/Figure S7.TIF]

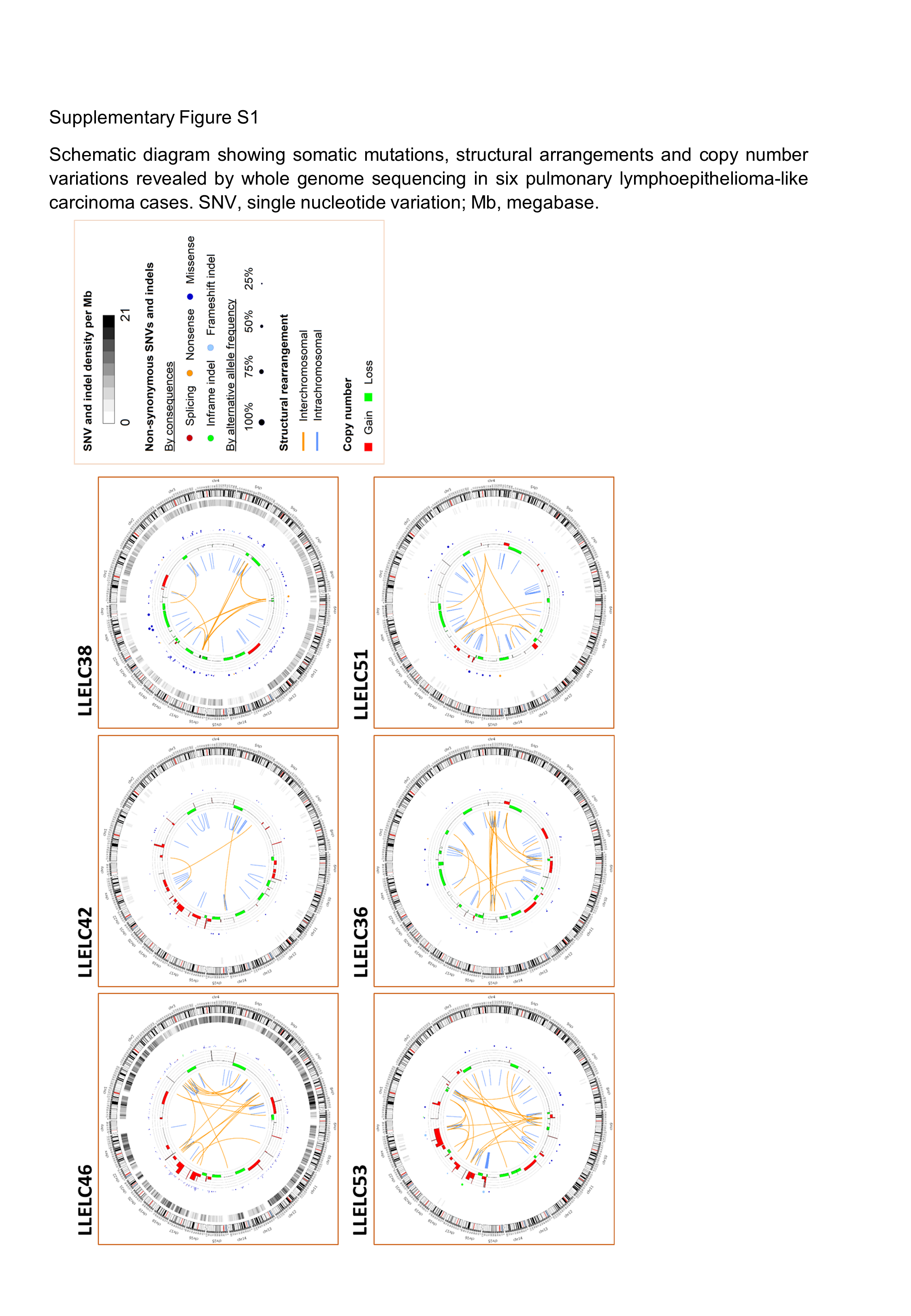

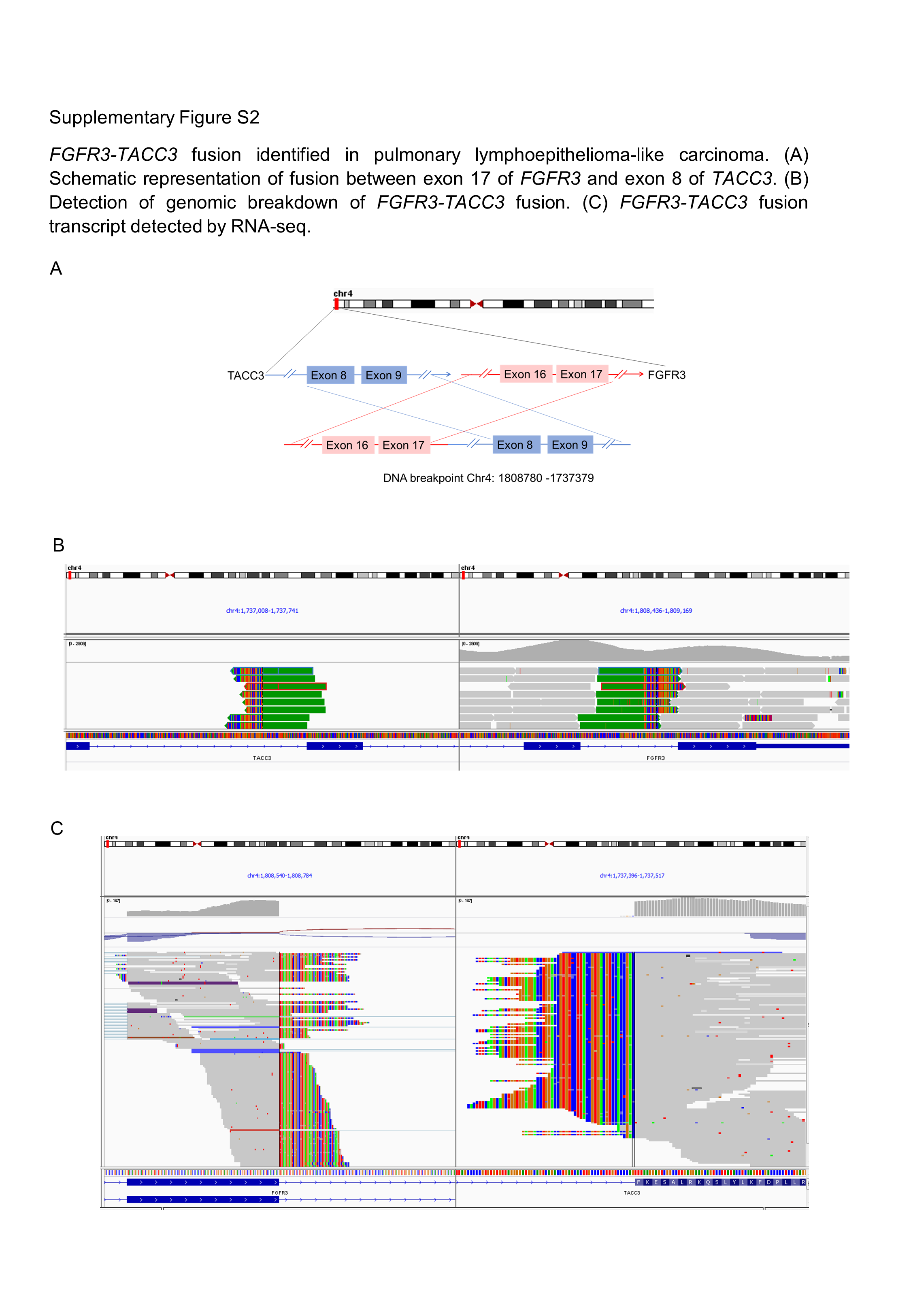

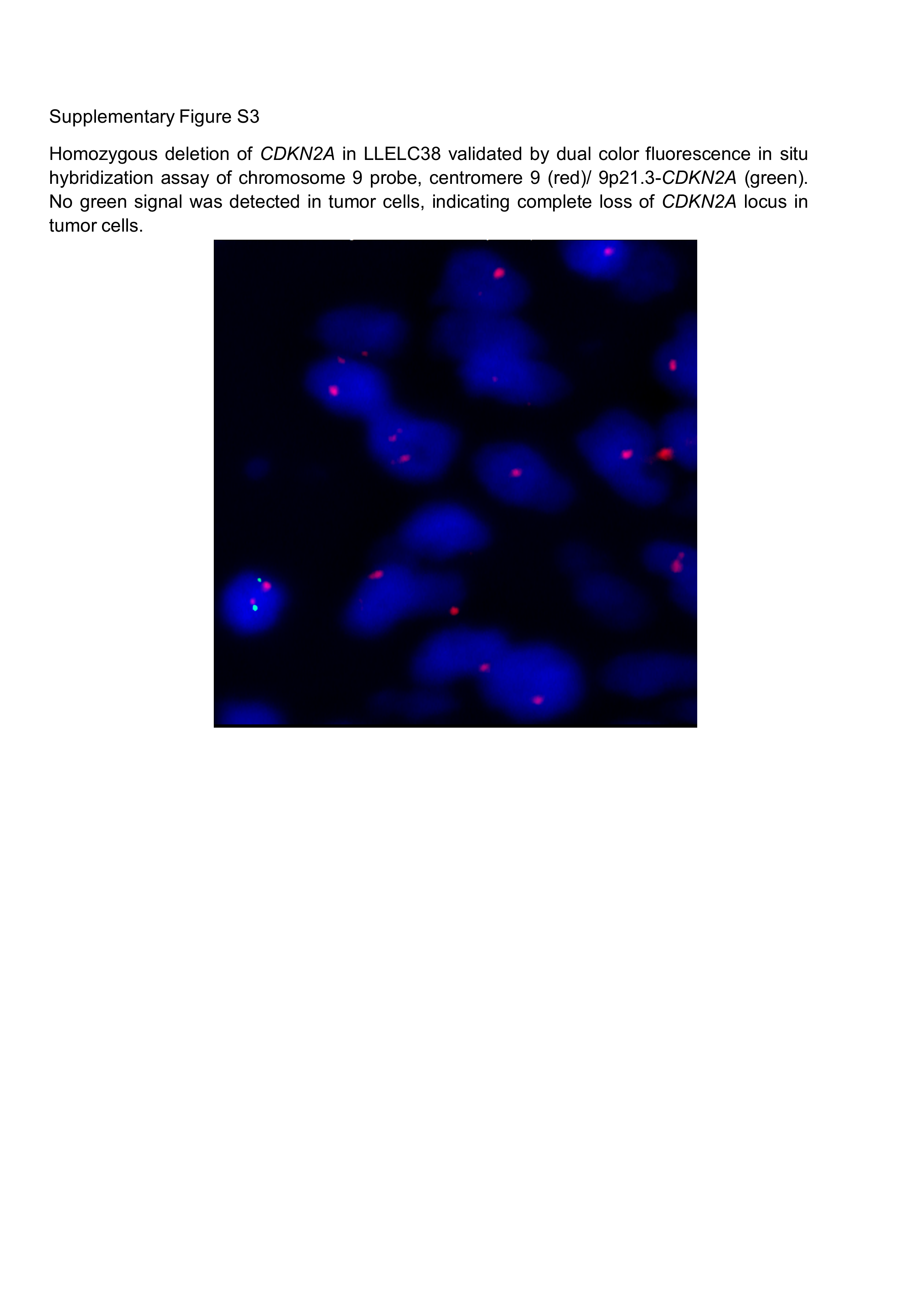

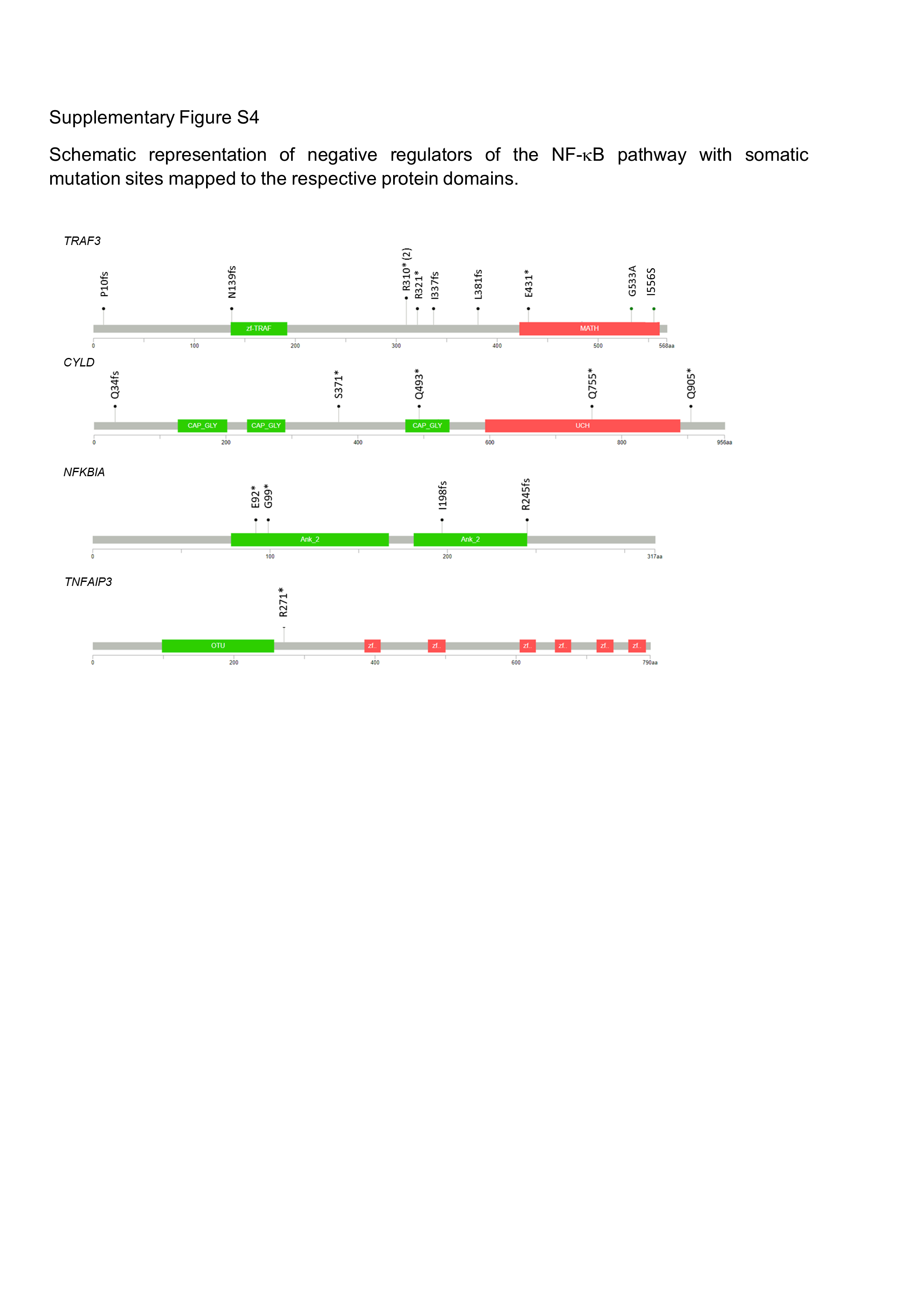

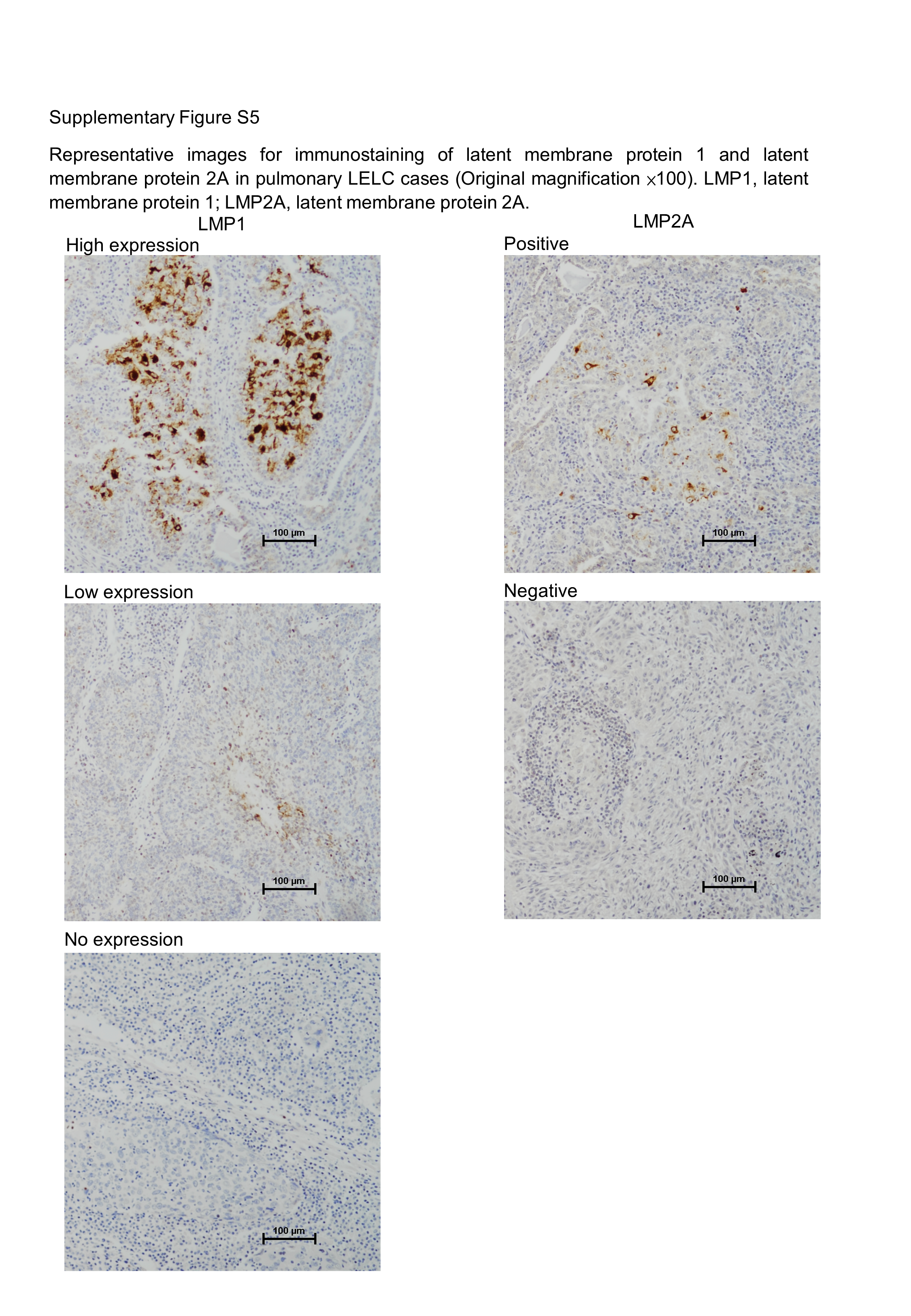

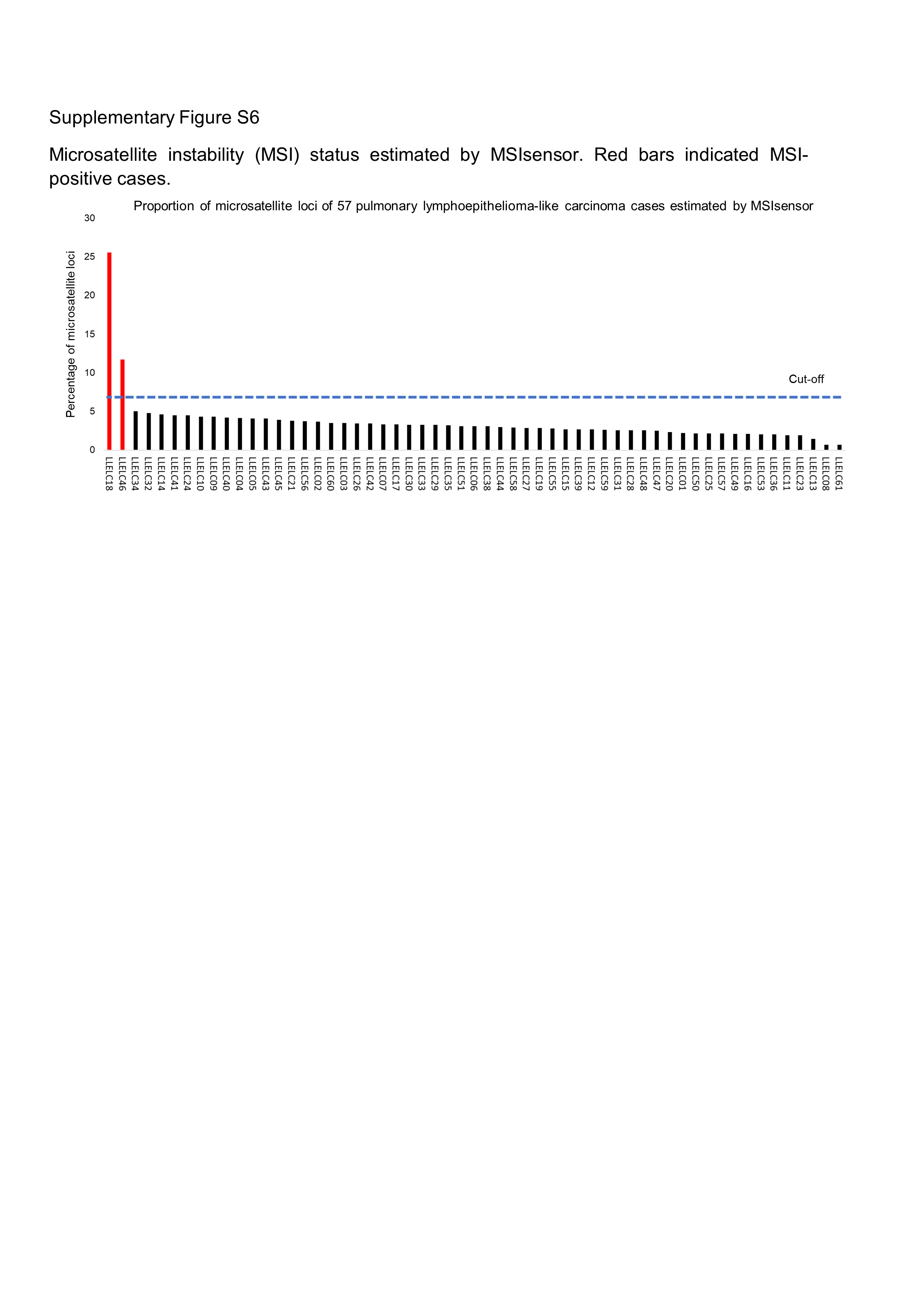

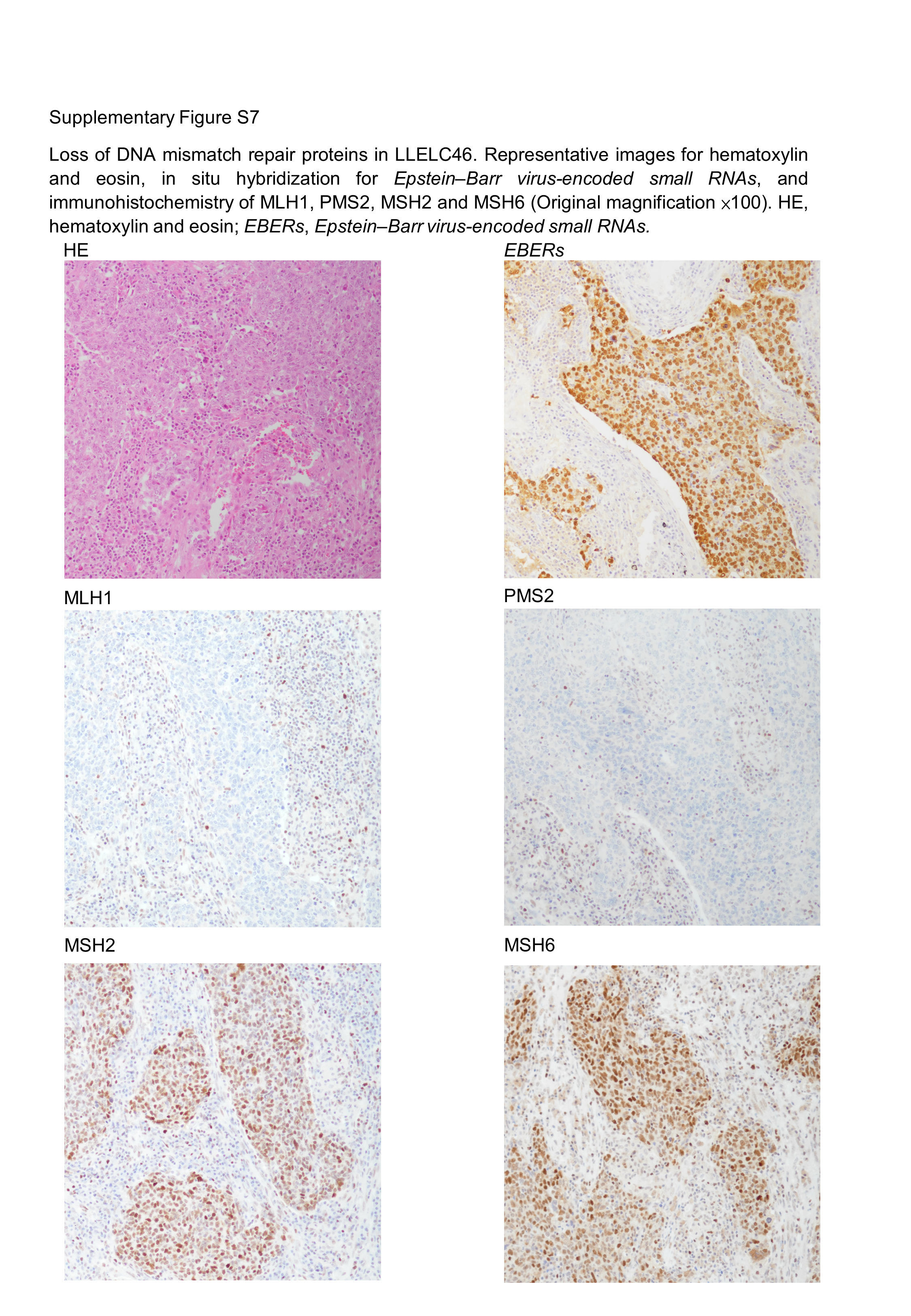

Supplement: Supplementary file 1 [file cancers-12-02065-s001.zip › cancers-870865-supplementary V2/supplementary figures.docx]
